# Towards in-field assessment of humeral and scapular kinematics: a comparison between laboratory and field settings using inertial sensors

**DOI:** 10.3389/fspor.2024.1349570

**Published:** 2024-02-28

**Authors:** Jackson Lordall, Opeyemi Vincent Akinluyi, Angelica E. Lang

**Affiliations:** Canadian Centre for Rural and Agricultural Health, College of Medicine, University of Saskatchewan, Saskatoon, SK, Canada

**Keywords:** wearable sensors, shoulder, home, work, sport, humeral elevation, humeral axial rotation, scapular upward rotation

## Abstract

**Introduction:**

Inertial measurement units allow for quantitative assessment of body motion in many environments. Determining the ability to measure upper limb motion with inertial measurement units, leveraging procedures traditionally used in the lab such as scapular calibration procedures and humeral axial rotation calculation, would expand the opportunities to assess upper limb function in externally valid environments. This study examined if humeral and scapular motion measured in different field settings is consistent with motion measured in a lab setting in similar tasks.

**Methods:**

Twenty-eight adults participated in the study (14 field setting, 14 lab setting). Three different types of field settings were included: home (*n* = 5), work (*n* = 4), and sports (*n* = 5). Field participants were matched to lab participants based on sex and body height. All participants were equipped with five inertial and magnetic measurement units (Xsens Awinda, Xsens Technlogies, NL, Fs = 100 Hz) on the torso, humeri, and scapulae. Humeral and scapular angles were measured during a functional task protocol consisting of seven tasks. Data from all three field settings were combined. Statistical parametric mapping (*α* = .05) was used to assess differences in waveforms between the lab and field data.

**Results and discussion:**

Five out of seven tasks displayed no differences for humeral elevation and humeral axial rotation, while scapular upward rotation and tilt were not statistically different for any tasks. Scapular internal rotation variability was very high for the field setting, but not for the lab setting. Task-based differences in humeral elevation and humeral axial rotation may be related to equipment modifications for the field protocol and between subjects' variability in task performance. Data indicate that humeral elevation, humeral axial rotation, and scapular upward rotation can be measured in externally valid field settings, which is promising for the evaluation of upper limb movement in natural environments.

## 1 Introduction

Quantitative scapular and humeral motion assessments are required to understand shoulder movement and upper limb abilities. Scapular and humeral motion are associated with shoulder injury risk (1–3), and shoulder injuries are prevalent in both the general population (4, 5) and athletic populations (6–8), necessitating further research into how the scapulae and humeri move in daily life. Inertial and magnetic measurement units (IMMUs) present a unique opportunity for quantitative assessment of scapular and humeral motion during functional movements in real-world settings, such as at work or during sport. However, there is a research gap regarding the use of IMMUs to measure humeral and scapular motion outside of the research laboratory. While some in-field work-specific assessments of posture or kinematics have been done for the upper arm (9–12), these assessments generally do not include humeral axial rotation or scapular motion, despite their importance to upper limb function and injury (13). Indeed, even in research lab settings, healthy and pathological scapular kinematics are most frequently measured during planar arm elevation (2, 14–20). To our knowledge, there exists no in-field measurement of the scapula during functional tasks, and humeral assessments are almost solely focused on arm elevation exposures (9–12, 21). Improved measurement of upper limb postures, including externally valid in-field assessments, could better inform interventions to prevent or address upper limb musculoskeletal disorders.

Best practices for IMMU use for measuring the humeri and scapulae during lab-based planar arm elevation have been previously defined (22–24). Recent research from our research group has validated the use of IMMUs for measuring both scapular movement (25) and humeral axial rotation (26) during a range of functional and work-related tasks. However, these studies have been conducted exclusively in lab settings, despite the portability of the IMMUs and their potential for in-field applications. These procedures differ from typical in-field motion capture set-up or analysis (27) due to external calibration tools for scapular tracking (23, 25) and calculations of rotation about the long axis of the arm (25, 28), which may be difficult to implement with field work. IMMUs can also be prone to drift or magnetic disturbance (29, 30); therefore, identifying if IMMUs and the necessary measurement and analysis protocol for measuring humeral and scapular motion can be applied to different field settings is necessary to confirm the utility of these methods or to identify potential sources of error that need to be addressed.

The primary objective of this study was to determine how humeral and scapular motion measured with IMMUs in different field settings (home, work, sport) compares to motion measured in a lab setting (26, 31). It was anticipated that the IMMUs will be able to successfully and accurately measure humeral and scapular kinematics in all in-field settings, as demonstrated by no difference in kinematic outcomes, as well as similar shape and magnitude of waveforms, between the field settings and the lab setting.

## 2 Methods

### 2.1 Settings

Three settings were used to test the in-field application of IMMUs for humeral and scapular motion capture: work, home, and sport. These settings were chosen to represent different areas of in-field motion capture application. For the work setting, data were collected in workshops or garages at farms or acreages. For the home setting, data were collected in participants' homes. For the sport setting, data were collected at a local outdoor baseball diamond. It is possible that different settings could have different environmental disturbances or practical data collection challenges, necessitating the testing in more than one field setting. For example, the work setting may cause more sensor disturbance from the surrounding equipment and tools, while the sport setting could present practical challenges regarding consistent sensor placement and computer operation due to the elements (i.e., wind, sun, etc). For the lab setting, participants were required to travel to the research lab on the University campus for data collection. Further detail about the lab sample can be found in previous research (26, 31). Participants from the lab dataset were selected and matched to participants in field settings based on sex and body height (±5 cm).

### 2.2 Participants

To be eligible for participation, prospective participants needed to be between the age of 18 and 65 years and free from any self-reported upper limb impairments such as shoulder pain. As this study was part of a larger protocol, additional inclusion criteria were required for the work and sport settings. Participants for the work setting were required to live or work on a farm or acreage in the area surrounding the University community. Participants for the sports setting needed to be actively participating in a throwing sport (e.g., baseball). The study protocol received approval from the institutional research ethics board. All participants provided written informed consent prior to participation.

### 2.3 Protocol

**Figure 1:**
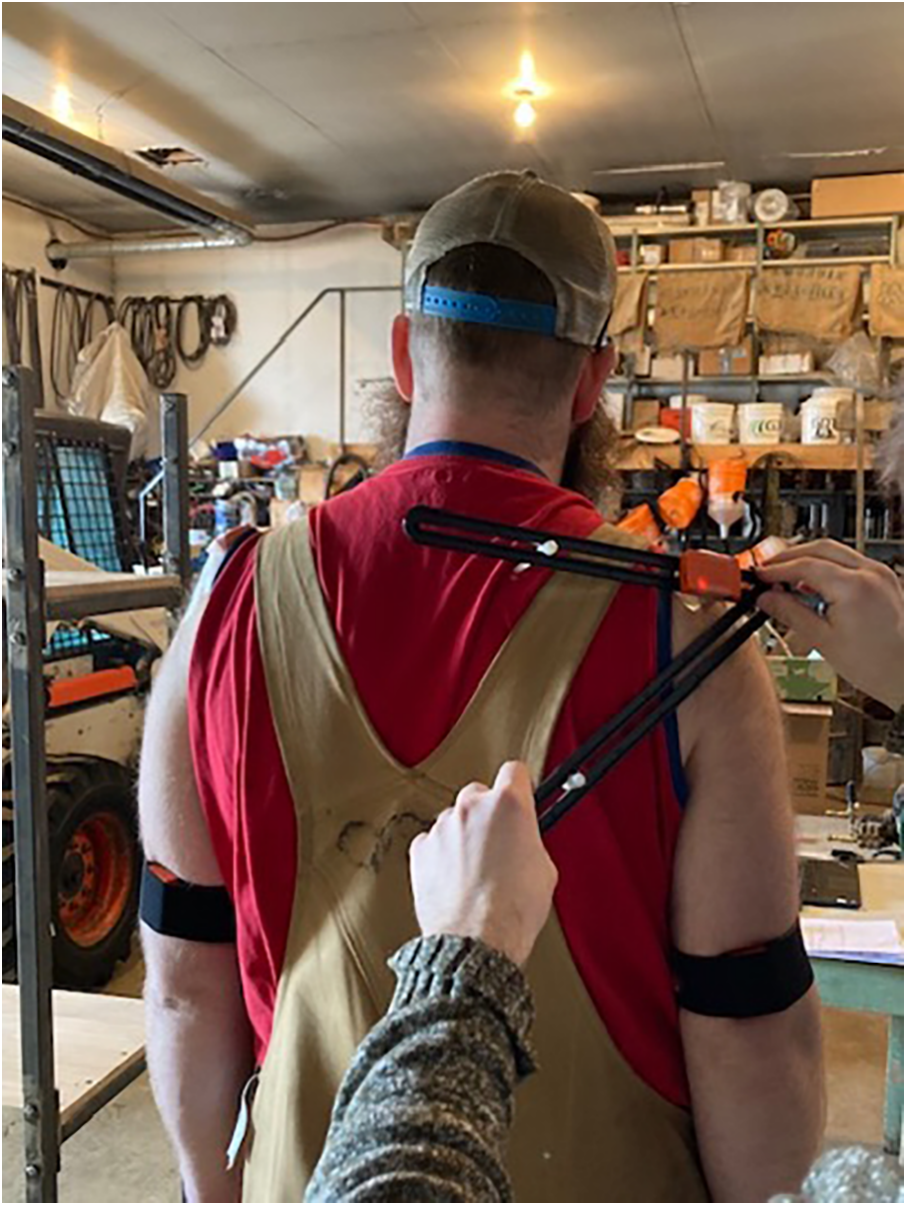
The locator used for the double scapular calibrations.

Participant demographic information, including biological sex, age, body mass, body height, and handedness, were recorded. Participants completed the Quick Disability of the Arm, Hands, and Shoulder (QuickDASH) questionnaire to assess upper limb abilities. Participants were equipped with inertial and magnetic measurement units (Xsens Awinda, Xsens Technlogies, NL, Fs = 100 Hz) on the flat part of the acromion of the scapulae, the posterior and distal aspects of the humeri halfway between the epicondyles, and the manubrium of the sternum based on previous research (22, 25). Bilateral scapular calibrations for the double calibration method (23, 25, 32) were completed by aligning a locator (23) (Figure 1) with the anatomical points of the scapulae to define the scapular orientation in a neutral position and at maximum humeral elevation. This calibration process is described in detail elsewhere (23, 25, 31). IMMU placements and calibrations were conducted by the same researcher across all field participants. For the lab setting, a different researcher from the field settings conducted all of the IMMU placements and calibrations across lab participants.

**Figure 2:**
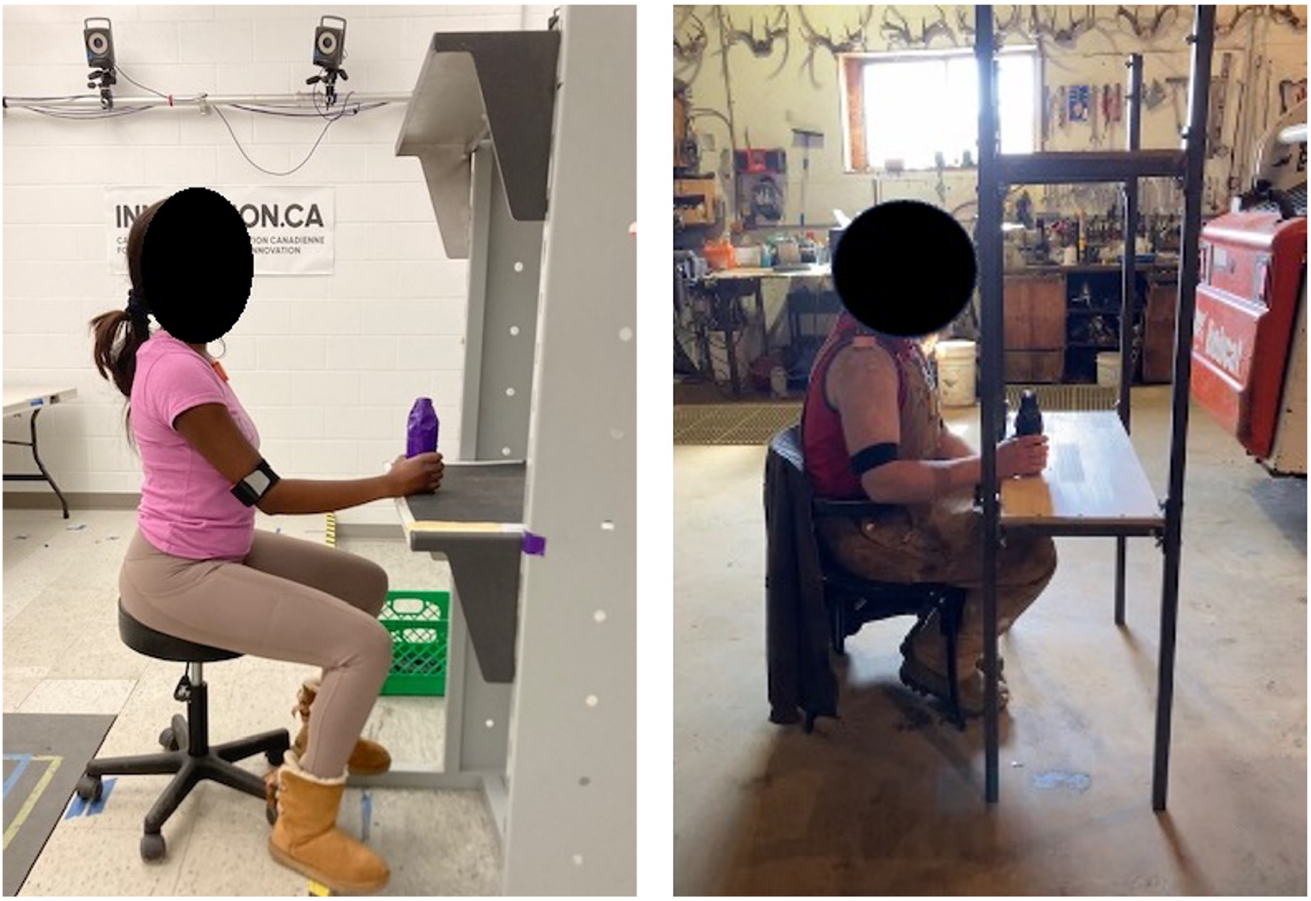
Lab setting (left) and field setting (right) shelf set up and start positions for overhead reach. For the lab setting, shelf heights for the lower and upper shelf were 75 cm and 143 cm, respectively. For the field settings, shelf heights were 75 cm and 150 cm. Participants were further away from the bottom shelf in the lab setting due to equipment positioning.

All participants completed the same functional task protocol called the Work-Related Activities and Functional Task (WRAFT) protocol to allow for direct comparisons between settings. The WRAFT protocol is an established lab protocol, which consists of several functional tasks that require participants to move their arms in many different planes and directions, encompassing many of the possible movements required during daily life (Table 1) (26). Specific start and end positions varied with task. Start positions were either arms by sides (Tie Apron, Floor Lift), hand resting on lap (Comb Hair, Wash Axilla), or hand resting on shelf or table (Overhead Reach, Forward Transfer, Overhead Lift). End positions are displayed in Table 1. Data for a Side Reach task typically included in the WRAFT protocol was not collected as previous work has found this task to have poor repeatability and high measurement error (26). We attempted to replicate the WRAFT protocol tasks in the field settings with the resources available and a custom-made shelf that was transportable and easy to set up. However, the shelf set ups were slightly different between the lab and field settings. In the laboratory, the distance between the data collection platform and the shelves prevented participants from sitting or standing directly in front of the bottom shelf. In contrast, participants in the field settings were able to sit or stand closer to the shelves, resulting in slightly different starting positions for the Overhead Reach, Forward Transfer, and Overhead Lift (Figure 2). Additionally, the Forward Transfer task was completed using available tables in field settings or by reaching forward to touch an imaginary tabletop if a table was not present. For all participants, three trials of each WRAFT task (three trials per arm for the unilateral tasks, three trials for bilateral tasks) were completed. For both the lab and field settings, each data collection session was less than 45 min in duration.

**Table 1:** Work-related activities and functional task (WRAFT) protocol tasks.

Task	Task description^a^
Comb hair	In a seated position with a comb, raising comb to forehead level and performing front to back combing motion
Wash opposite axilla	In a seated position, raising a washcloth to opposite axilla and performing washing motion
Tie apron	Pantomiming tying an apron behind the back
Overhead reach	Lift a 1 kg bottle onto a 1.5 m tall shelf
Forward transfer	Move a 1 kg bottle approximately 50 cm in the forward direction
Floor to waist lift	Lift a milk crate (8 kg) from the ground onto a shelf at waist height
Overhead lift	Lift a milk crate (8 kg) from approximately waist height to a shelf overhead

^a^ For more details, see Friesen et al. (26).

### 2.4 Data analysis

Rotation matrices were exported from XSENS MT Manager^TM^, and scapular and humeral angles were calculated using custom Matlab® scripts. Humeral elevation was calculated as the angle between the long axes of the torso and humerus as this is the most consistent method to define humeral elevation. Humeral axial rotation was calculated using the True axial rotation method (26, 28, 33). The True axial rotation method is calculated by numerically integrating the humeral angular velocity vector projection onto the humeral longitudinal axis (28) and has been determined to be the most reliable method for calculated axial rotation using different motion capture methods (33). Scapular angles (internal rotation, upward rotation, and tilt) were calculated as Euler angles using the YXZ sequence (26, 34). Trials within each task and participant were averaged and used for the analysis. Scapular and humeral angle waveforms were plotted over defined movement cycles starting at 5 degrees of humeral movement to peak humeral position (33).

### 2.5 Statistical analysis

Data from all three field settings were averaged to improve statistical power, as the preliminary analysis indicated that the data collected in the different field settings were not statistically different. Differences in demographic characteristics between the field and lab groups were tested using an independent samples *t*-test (*α* = .05) for height and Mann–Whitney *U* tests (*α* = .05) for mass, age, and QuickDASH scores. For each WRAFT protocol task, humeral and scapular angle waveforms from the field settings were compared to waveforms in the lab descriptively and with statistical parametric mapping (SPM) (35, 36) (*α* = .05) in Matlab®. SPM was chosen to compare these two different groups, as it can compare full waveforms to assess magnitude and temporal factors. Additionally, because variability between different datasets can influence results, standard deviation for each angle waveform was compared between settings using Mann–Whitney *U* tests (*α* = .05). Traditional agreement or repeatability measures are not reported as this was not a test-retest study design.

## 3 Results

Twenty-eight adults (14 in-field, 14 in-lab) participated in the study (Table 2): five in the home setting, four in the work setting, five in the sport setting, and fourteen in the lab. There were no significant differences in age, mass, height, and QuickDASH scores between field participants and lab participants (*p* > .05) (Table 2). Additional data from two participants collected in the field settings (one home, one work) (not included in the above group of 14 participants) were not incorporated into the analysis due to calibration and collection errors, respectively.

**Table 2:** Demographics of study participants.

	Field settings (*n* = 14)	Lab setting (*n* = 14)	*p*
Sex (male:female)^a^	9:5	9:5	–
Handedness (right:left)^a^	13:1	14:0	–
Age (years)^b^	31 ± 13	29 ± 12	.511
Mass (kg)^b^	74 ± 19	80 ± 24	.667
Height (cm)^b^	172 ± 15	171 ± 13	.444
QuickDASH score^b^	3 ± 4	1 ± 2	.210

^a^ Data are provided as counts.

^b^ Data are provided as mean ± standard deviation.

### 3.1 Humeral elevation

**Figure 3:**
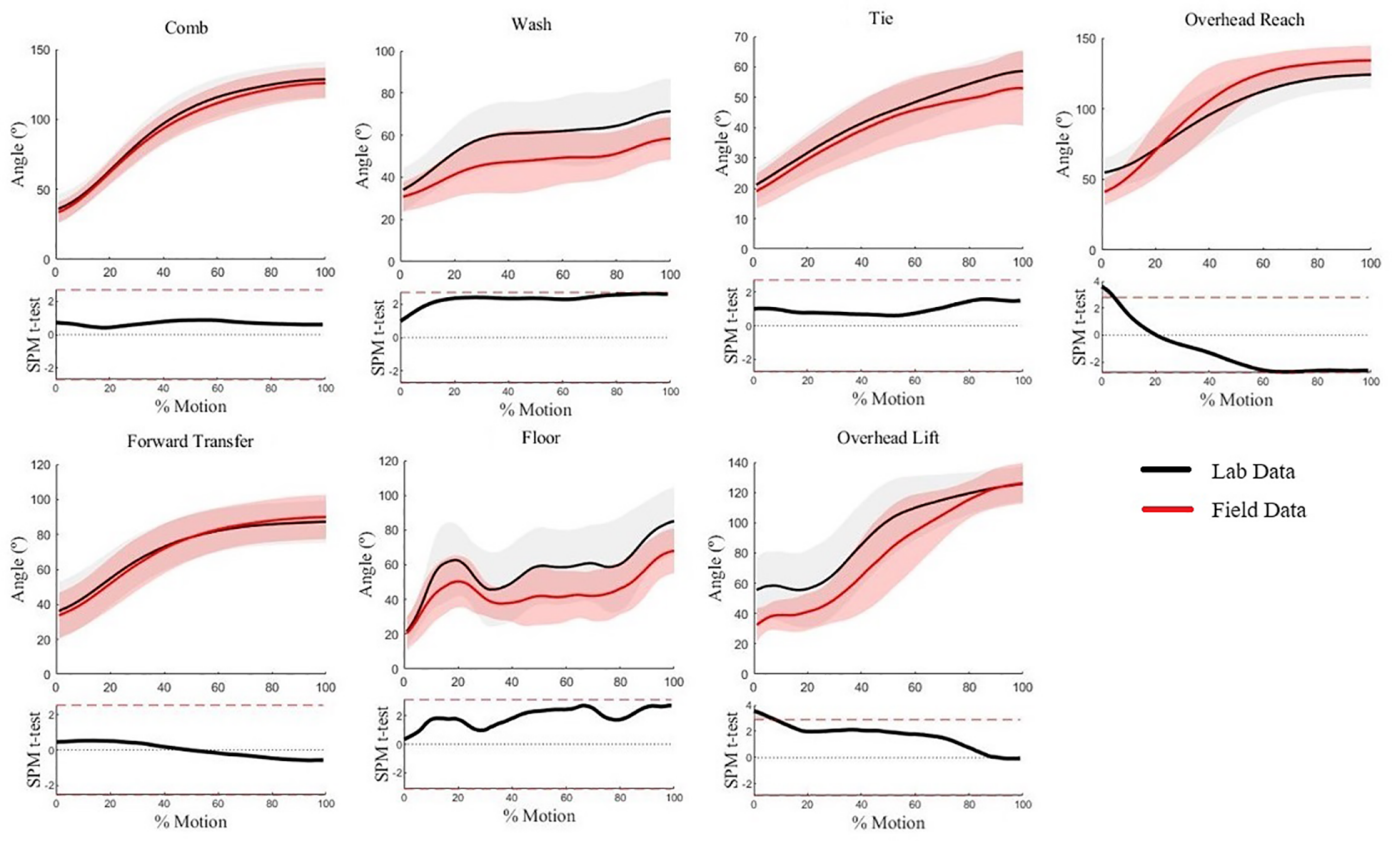
Humeral elevation waveforms for each WRAFT protocol task performed in the field settings (red) and the lab setting (black). Bold lines represent mean waveforms, shaded areas represent one standard deviation. The corresponding statistical parametric mapping test comparing waveforms between the field and the lab setting are provided below the waveform plot for each task.

Humeral elevation waveforms for five of seven WRAFT protocol tasks were not statistically different between the field and lab (*p* > .05) (Figure 3). Statistical differences in humeral elevation magnitudes (max mean difference = 20°) were present for Overhead Reach and Overhead Lift only at the very beginning of the tasks (0%–6%), with greater humeral elevation in the lab setting (*p* < .05) (Figure 3). Average standard deviations were similar between settings (14.1° in lab vs. 13.7° in field, *p *= .70).

### 3.2 Humeral axial rotation

**Figure 4:**
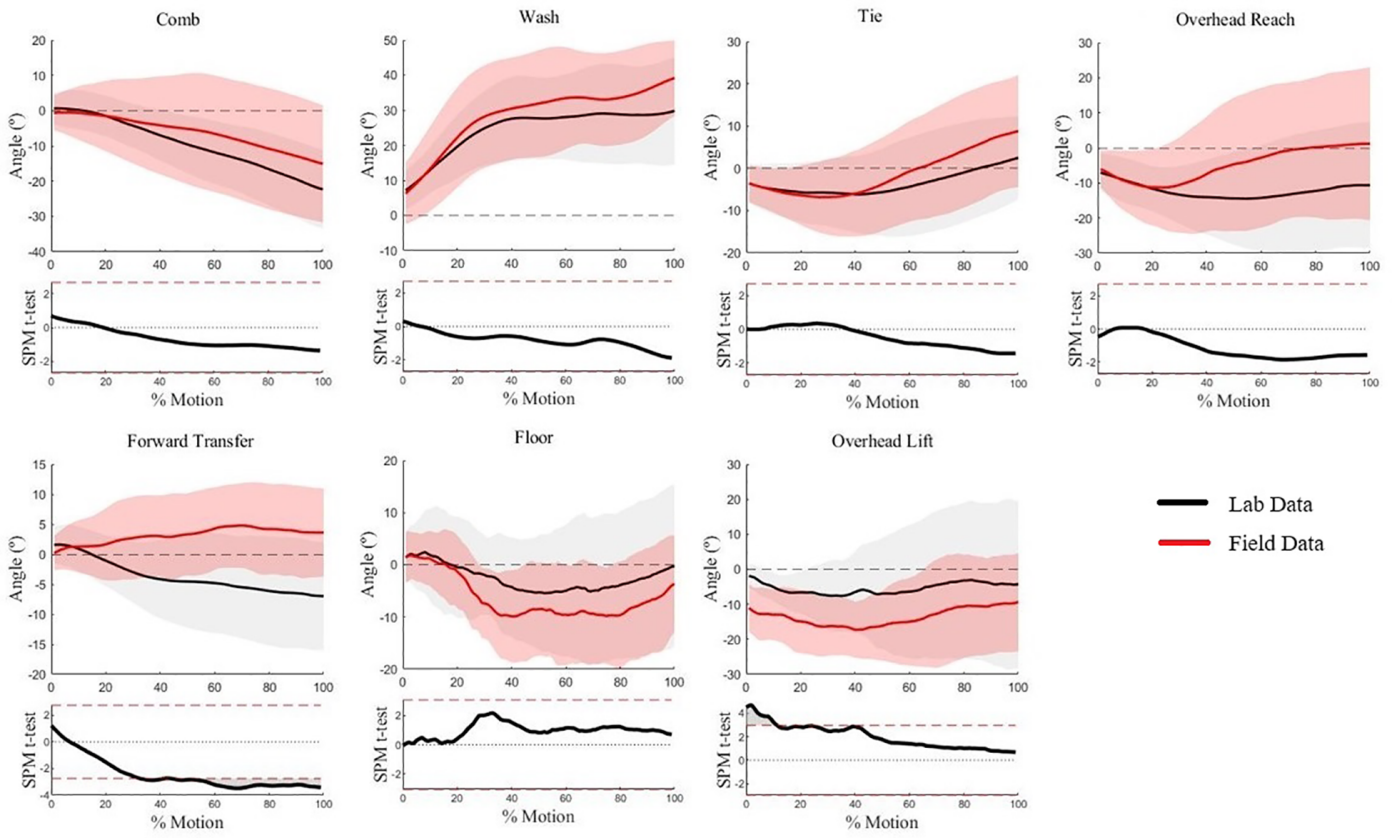
Humeral axial rotation waveforms for each WRAFT protocol task performed in the field settings (red) and the lab setting (black). Bold lines represent mean waveforms, shaded areas represent one standard deviation. The corresponding statistical parametric mapping test comparing waveforms between the field and the lab setting are provided below the waveform plot for each task.

Humeral axial rotation waveforms for five of seven WRAFT protocol tasks were not statistically different between settings (*p* > .05) (Figure 4). There was greater humeral external rotation in the lab setting when compared to the field settings between 60%–100% of the Forward Transfer task (max mean difference = 11°, *p* < .05) (Figure 4). There was greater humeral external rotation in the field settings compared to the lab setting at the beginning of the Overhead Lift task (max mean difference = 9°, *p* < .05) (Figure 4). Average standard deviations were similar between settings (9.1° vs. 10.7°, *p* = .33).

### 3.3 Scapular upward rotation

**Figure 5:**
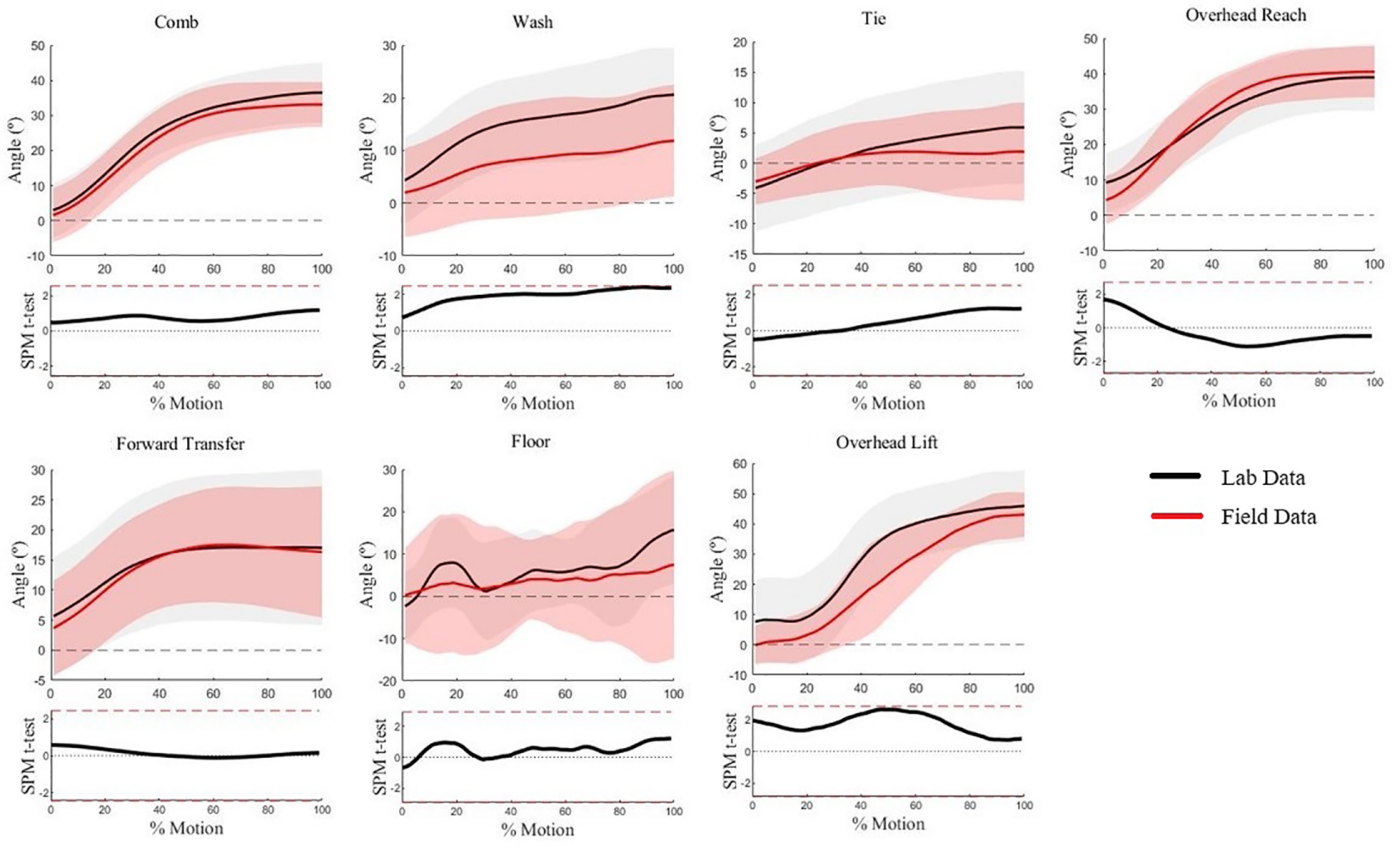
Scapular upward rotation waveforms for each WRAFT protocol task performed in the field settings (red) and the lab setting (black). Bold lines represent mean waveforms, shaded areas represent one standard deviation. The corresponding statistical parametric mapping test comparing waveforms between the field and the lab setting are provided below the waveform plot for each task.

Scapular upward rotation waveforms for all WRAFT protocol tasks were not statistically different between settings (*p* > .05) (Figure 5), and average standard deviations were similar across tasks and settings (10.3° vs. 12.9°, *p *= .17). Small magnitude differences were evident from visual analysis for Wash Axilla and Overhead Lift, but these differences did not reach statistical significance (Figure 5).

### 3.4 Scapular tilt

**Figure 6:**
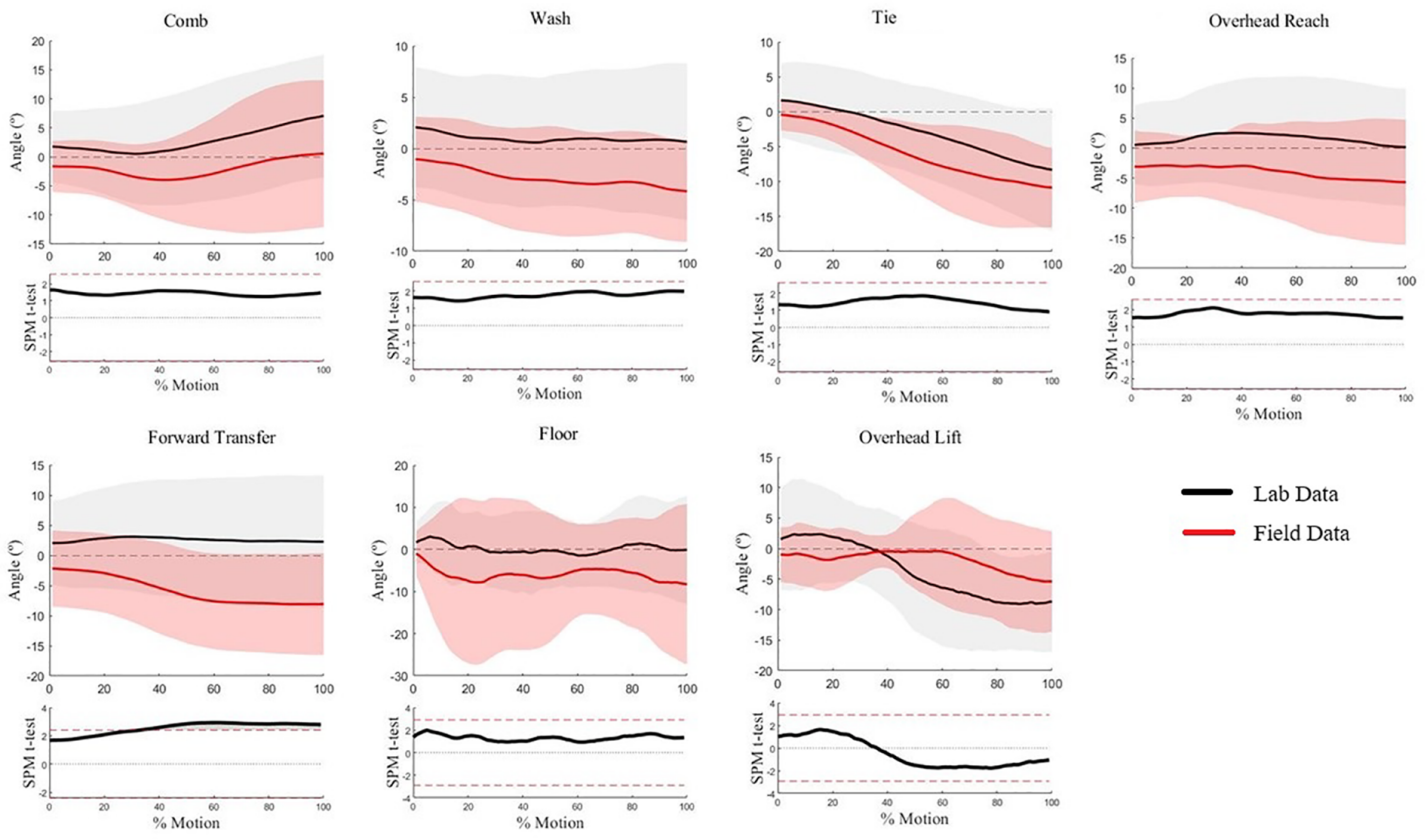
Scapular tilt waveforms for each WRAFT protocol task performed in the field settings (red) and the lab setting (black). Bold lines represent mean waveforms, shaded areas represent one standard deviation. The corresponding statistical parametric mapping test comparing waveforms between the field and the lab setting are provided below the waveform plot for each task.

Scapular tilt waveforms were not statistically different between settings (*p* > .05) (Figure 6). A consistent magnitude offset of approximately 5° is visually present for many tasks, but waveform trajectory is similar for all tasks expect for the overhead lift. However, average standard deviations were significantly higher for the field settings (8.0° vs. 16.3°, *p *= 0.017).

### 3.5 Scapular internal rotation

**Figure 7:**
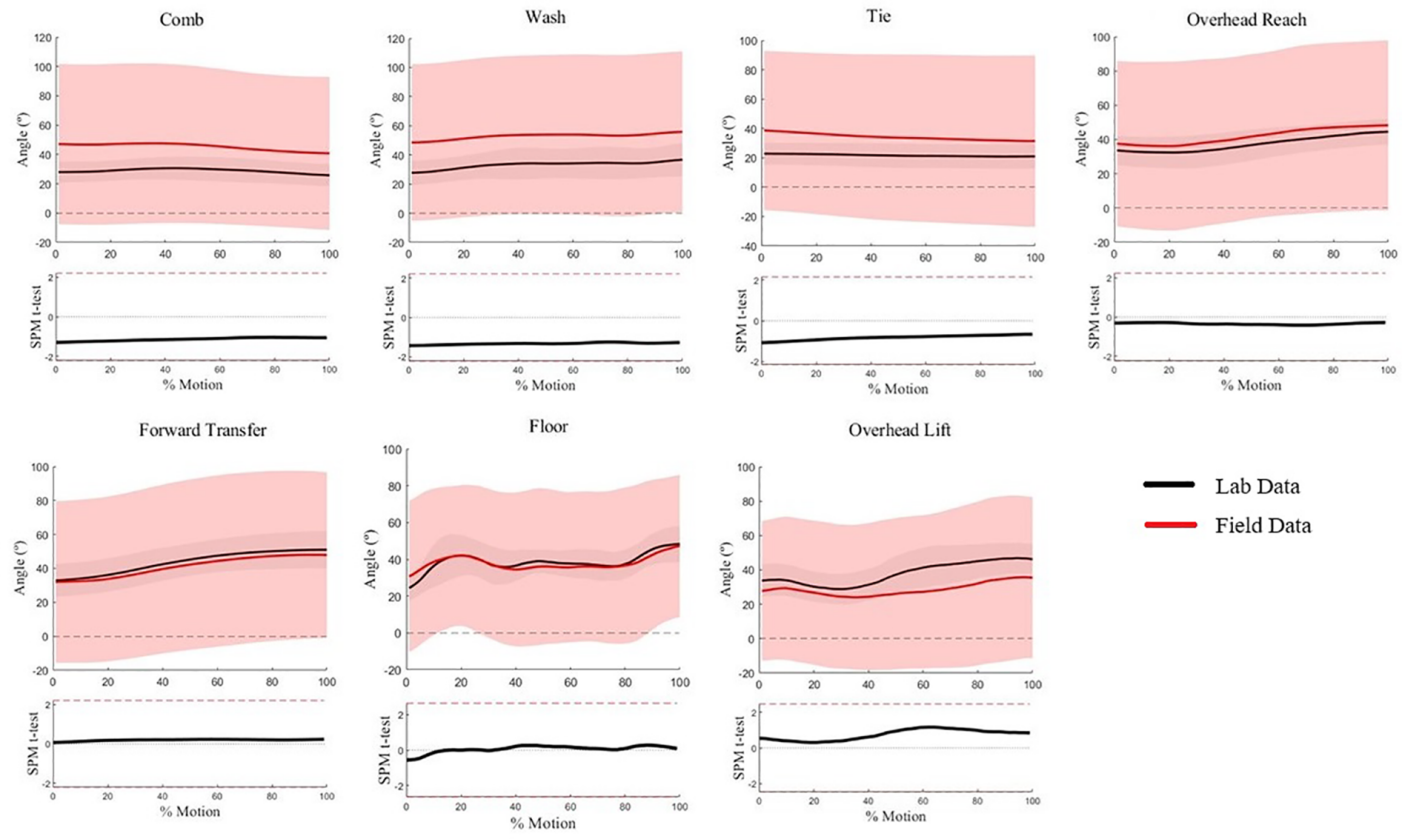
Scapular internal rotation waveforms for each WRAFT protocol task performed in the field settings (red) and the lab setting (black). Bold lines represent mean waveforms, shaded areas represent one standard deviation. The corresponding statistical parametric mapping test comparing waveforms between the field and the lab setting are provided below the waveform plot for each task.

While scapular internal rotation waveforms displayed no significant differences between settings (*p* > .05) (Figure 7), the standard deviation of the field setting (the shaded light pink area on Figure 7) is substantially and significantly larger than the lab setting (22° vs. 76°, *p *= .002).

## 4 Discussion

The current study examined whether humeral and scapular kinematic data collected using IMMUs in various field settings (home, work, sport) is comparable to IMMU data collected in a lab setting. Findings indicate that humeral elevation, humeral axial rotation, scapular upward rotation, and, to some extent, scapular tilt measured in-field were not different from data collected in a controlled lab setting. Field data were collected in different home, work, and sport settings suggesting that these methods can be applied for in-field research and may be robust and generalizable across settings. Researchers can begin to use these methods to assess upper limb motion in natural environments, such as sporting arenas or workshops, which may better inform understanding of upper limb musculoskeletal disorders. The ability to capture natural movements, whether in sport, work, or daily life activities, will have positive implications for detailed investigations of how biomechanics influences performance and injury risk.

Given the similarity of humeral waveforms for most WRAFT protocol tasks, some of the observed differences in upper limb motion may be explained by equipment modifications required to implement the protocol in the field settings (37–39). Different set ups between the lab and field settings (Figure 2) may explain greater humeral elevation and external rotation in the lab at the beginning of the Overhead Reach and Lift tasks. Greater humeral elevation magnitudes at the beginning of the Overhead Reach and Overhead Lift tasks may be due to different start positions in the lab and field settings. In the lab setting, participants were not able to sit or stand as close to the shelf in the lab setting as compared to the field settings. Greater humeral external rotation for the Forward Transfer task in the lab setting compared to the field settings may also be due to differences in task set up: in the lab, all participants sat at the same table, while in the field setting, participants either sat at a table that was available in that setting or sat in front of the set of shelves and reached beyond to an “imaginary” table top. The observed differences in upper limb motion are explainable, suggesting that the observed differences may be largely due to equipment or set up modifications and not the measurement system.

The between-subjects' design may have contributed to visual differences in waveform magnitude for scapular upward rotation and tilt outcomes and increased variability in tilt. Between-subjects' variability was considered by matching field participants to lab participants by sex and body height, but because the comparisons are of two different samples, individual variation likely affected the results (40, 41). For scapular tilt, the larger standard deviations for the field data could mask significant differences; therefore, caution should be used when interpreting those results.

The upper limb movements during WRAFT protocol tasks were relatively unconstrained, allowing for self-selected movement patterns following standardized verbal instruction and visual demonstration of each task. Increasing task constraints, such as movement speed (42, 43), could have further minimized the differences between settings. Despite this variation, humeral elevation, humeral rotation, and scapular upward rotation demonstrate minimal differences, similar visual movement trajectories, and similar variability between settings and to previously reported data (i.e., 10°–24°) (16, 31, 36, 44–47). As between-groups comparisons are prevalent in biomechanics, the similarity of the mean and standard deviation of angle waveforms between the lab and field datasets is promising. However, a test-retest study design would increase the robustness of the findings and interpretation.

Scapular internal rotation measurement was not successful in this study. Significant differences are not present due to the very wide standard deviations for the in-field measures, demonstrating the inconsistency of the scapular internal rotation measurements. There may be two primary explanations for this issue: (1) IMMU movement on the skin, and (2) distortion of the IMMUs. First, in the initial lab-based validation of the scapular IMMU measurement methods (25), the tracking IMMU was placed directly on, and likely braced by, the rigid, L-shaped motion capture acromial marker cluster. In the field, the IMMU was placed directly on the skin above the flat part of the acromion (25, 48, 49). Without the support of the motion capture cluster, the tracking IMMU could have moved on the skin around the *Y* axis, affecting internal rotation outcomes. Second, it is documented that magnetic distortion of IMMUs largely affects rotation about the *Y* axis (29, 50), which corresponds with scapular internal rotation in this set-up. The error levels in this previous research are considerably lower than the variability in the current study; it is possible that any distortion affects could be compounded as the scapular angle calculations rely on three separate IMMUs (locator, sternum, scapular tracking IMMU). These same issues are not present in the humeral axial rotation, which is traditionally calculated as rotation about the humeral *Y* axis (34, 51), because the current study leverages the newer “True axial rotation” method, which does not rely on Euler angles. Overall, it may not be possible to accurately analyze scapular internal rotation with the current set-up. Future research will further investigate possible distortion affects and mitigation strategies.

There are some limitations to address with this study. Although we attempted to make the lab and field protocols identical, there were differences in equipment and protocol between lab and field groups. While our study demonstrates that good quality data can be collected over periods of approximately 45 min, it is unclear whether results can be extended to data recording sessions spanning over several hours. Additionally, data may not generalize across all outdoor and indoor settings, as data could differ from what is reported in settings with local magnetic field disturbances. Future research should consider the specific environment when measuring movement with IMMUs. Importantly, due to the between subjects' design, we were not able to use traditional agreement, repeatability, or reliability tests such as Bland-Altman plots, intraclass correlation coefficients, or standard error of measurement. However, as the purpose was to assess if the setting affected IMMU kinematic outputs, the lack of differences for many angles, and the similarity of many waveforms, even for these two separate (matched) datasets, we believe our approach and conclusions are valid. Future research will explicitly explore agreement between settings using a test-retest design with the same participants. Finally, as mentioned scapular internal rotation did not demonstrate acceptability similarity to the lab data. Scapular angles were calculated from Euler angles, suggesting angle calculation crosstalk could also affect upward rotation and tilt, although findings suggest these degrees of freedom were minimally affected. Further work is needed to understand and address this issue.

These findings provide a foundation for future research with IMMUs. Beyond the methodological work to refine these methods, as described above, the next steps should focus on applying these methods to measure real, daily life movements in each environment. Application of IMMUs in different field settings presents an opportunity for interdisciplinary research exploring how biomechanics, sport sciences, wearable technology, and clinical science can work together to address research gaps with modern solutions. Specifically, upcoming research will build on this work to assess sport-specific movements with IMMUs, such as overhead throws and different pitch types on baseball diamonds, or work-specific tasks, such as machinery maintenance and animal care on grain and large animal farms.

## 5 Conclusion

Humeral elevation and axial rotation and scapular upward rotation show promise for biomechanical assessments in different field settings using IMMUs with the methods used in this study. However, due to increased variability in field data, scapular tilt outcomes should be interpreted with caution and scapular internal rotation should not be assessed with IMMUs outside of the laboratory with these procedures. These results are encouraging for the evaluation of upper limb motion in natural environments.

## Data Availability

The raw data supporting the conclusions of this article will be made available by the authors, without undue reservation.
